# Supplementary material for: Co-development of an evidence-based personalised smoking cessation intervention for use in a lung cancer screening context
Source: BMC Pulm Med. 2022 Dec 15;22:478. doi: 10.1186/s12890-022-02263-w (PMC9756588; doi:10.1186/s12890-022-02263-w)
Supplement: Supplementary file 3 — Additional file 3. GRIPP2 Short Checklist. This file shows a completed GRIPP2 short reporting checklist. [file 12890_2022_2263_MOESM3_ESM.pdf]

Additional File 3. GRIPP short reporting checklist. From: GRIPP2 reporting checklists: tools to improve reporting of patient and public involvement in research

**Section and topic**	**Item**	**Reported on page No**
1: Aim	Report the aim of PPI in the study	4-6
2: Methods	Provide a clear description of the methods used for PPI in the study	8-9
3: Study results	Outcomes – Report the results of PPI in the study, including both positive and negative outcomes	9-12
4: Discussion and conclusions	Outcomes – Comment on the extent to which PPI influenced the study overall. Describe positive and negative effects	13-16
5: Reflections/critical perspective	Comment critically on the study, reflecting on the things that went well and those that did not, so others can learn from this experience	15-16
